# Syphilitic Phthisis in the Negro

**Published:** 1881-05-20

**Authors:** G. G. Roy

**Affiliations:** Professor of Materia Medica in the Southern Medical College


### SYPHILITIC PHTHISIS IN THE NEGRO.

BY G. G. ROY, M. D., PROF. OF MATERIA MEDICA IN THE SOUTHERN MEDICAL COLLEGE.

The development of phthisis pulmonalis in the negro race of the South since the late war, has become a serious question, and one of grave consideration—present and prospective—for the medical profession, as well as the thoughtful humanitarian.

From being an extremely rare occurrence to meet with this disease in the full-blooded negro before the war, the mortality among them from it is now appalling.

And as evidence of the fact that the causes of this increased development of the disease have arisen mainly since the war, the *young* are its chief victims—the ages ranging from 12 to 30 years. As one of the city's physicians for the last two years, my opportunities for studying the prevailing causes of mortality among the colored population—a large majority of which are paupers when disease of any kind overtakes them—have been unusually good.

Early after being installed into the position of Fourth ward physician, my record shows that in the space of a few weeks during the fall of 1879, burial at the city's expense was furnished for ten young negroes—aged from 12 to 30—who had died of consumption; and from that time to this, I have observed no decrease in the rate of mortality.

The important and interesting question was at once presented, to learn the causes of this unusual development of the disease and the mortality.

After very careful investigation, my observations convinced me that the two prominent causes were syphilis and imprudent exposure of the body to night air and inclement weather, after a night's carousal. I have no doubt the experience of the physicians of all our Southern cities bears testimony to the increased ravages of syphilis among the negro race since the war, though they may not have connected the increased development of phthisis with this agency.

The negro race, since "freedom," has become extremely migratory—very restless, never satisfied to remain long in a place, and ever on the alert for some new and rich pasture upon which to browse. In their wanderings many of them reach the larger cities, where they seem to find all of their long-sought joys, and where culminate all of their earthly ambition and brightest dreamed-of hopes. Unaccustomed to "city life and ways," he is soon introduced into all the haunts of vice and sin which afflict our cities. The sequel is, that even "before his suspicions are aroused," he has contracted syphilis, and soon scatters it broadcast, at home and among his numerous female acquaintances.

Of these cases not one in a hundred is ever under treatment long enough to have the disease eradicated. They generally fall into the hands of quacks, a la Buchanan, who pronounce them "cured" as soon as the primary lesion disappears, and they go on in ignorant bliss of their terrible fate.

The poison, the meanwhile lurking in the system, the disease passes into the secondary stage, and if phthisis and death do not intervene, the tertiary stage is reached with all of its life-long miseries. The irregular and exposed life of the average negro has a strong tendency to invite disease to the respiratory organs. The most sober-sided among them do not observe the ordinary hygienic rules of life.

They disregard entirely Franklin's law of eight hours rest or sleep, and prowl around all hours of night, in all weather, attending either the meetings of some one of their many "societies," their church or social gatherings; at all of these they commonly pass through feverish stages of animal excitement—dancing, shouting or exhorting; this is followed by complete relaxation and often prostration. In this physical and mental condition they wend their way homeward, their bodies often poorly protected by clothing, at the most chilling hour of the night, and ofttimes through the most inclement weather. Having reached home, without fire, and scant of bedding, they throw themselves down—frequently without removing their clothes—for a few hours' nap before they start out on their daily rounds of work or loafing.

The young are particularly addicted to this unhealthy mode of life.

Could there be more potent influences than these to develop or excite pulmonary disease in a subject already inoculated with the hydra-headed poison of syphilis? Are we not sadly in need of sanitary regulations to arrest, in a measure at least, the causes of this wholesale destruction of the usefulness and lives of a large class of our population? Much might be done, were judicious efforts made in the proper direction.

My impression is that syphilitic phthisis is a much more common disease than we have heretofore considered it, in both races, and the unhealthy habits and usage to which the negroes of our Southern cities are subjecting themselves by their irregular and dissolute mode of living, is the *chief* cause of that fearful mortality among them.

